# The Predictive Value of Clinical Signs to Identify Shock in Critically Ill Patients

**DOI:** 10.3390/diagnostics15172252

**Published:** 2025-09-05

**Authors:** Matthias Noitz, Sabine Preining, Dominik Jenny, Simon Langthaler, Romana Erblich, Thomas Tschoellitsch, Jens Meier, Martin W. Dünser

**Affiliations:** 1 Department of Anesthesiology and Critical Care Medicine, Kepler University Hospital GmbH, Johannes Kepler University Linz, Krankenhausstrasse 9, 4020 Linz and Altenberger Strasse 69, 4040 Linz, Austria; 2 Medical Faculty, Johannes Kepler University Linz, Altenberger Strasse 69, 4040 Linz, Austria

**Keywords:** lactate, radial pulse, capillary refill time, peripheral perfusion, skin mottling, shock index, clinical signs, shock

## Abstract

**Background/Objectives:** Current guidelines recommend the use of clinical signs to diagnose shock and cellular hypoperfusion in critically ill patients. However, these recommendations are based on limited scientific evidence. The objective was to determine the predictive value of clinical signs to identify shock. **Methods:** Retrospective cohort study including adult (≥18 years) patients admitted to the critical care resuscitation unit of a tertiary hospital. The primary goal was to determine the predictive value of tachycardia, prolonged capillary refill time (CRT), skin mottling, weak radial pulse, inadequate peripheral perfusion, shock index > 0.8, altered mental state, and diaphoresis to identify shock. Two-by-two contingency tables were used for statistical analysis. **Results:** Three-hundred-seventeen patients (no shock, *n* = 231; shock, *n* = 86) were included. As a single clinical sign, skin mottling [sensitivity, 0.38; specificity, 0.92; negative likelihood ratio (LR−), 0.68; positive likelihood ratio (LR+), 4.62], prolonged CRT (sensitivity, 0.44; specificity, 0.89; LR−, 0.62; LR+, 4.17), shock index >0.8 [sensitivity, 0.77; specificity, 0.64; LR−, 0.36; LR+, 2.15], a weak radial pulse [sensitivity, 0.62; specificity, 0.79; LR−, 0.49; LR+, 2.88], and inadequate peripheral perfusion [sensitivity, 0.68; specificity, 0.73; LR−, 0.44; LR+, 2.52] predicted shock. Prolonged CRT, skin mottling, inadequate peripheral perfusion, a weak radial pulse, and a shock index >0.8 predicted shock states with low cardiac output. A shock index >0.8, tachycardia, and a weak radial pulse were predictive of distributive/vasodilatory shock. The accuracy to identify shock were higher if ≥2 clinical signs were present compared to only one. **Conclusions:** Skin mottling, prolonged CRT, shock index >0.8, weak radial pulse, and inadequate peripheral perfusion can identify patients with shock, particularly shock states with low cardiac output, with high specificity and LR+.

## 1. Introduction

The term shock refers to a life-threatening, generalized form of acute circulatory failure associated with inadequate cellular oxygen utilization and increased blood lactate levels [1,2]. Shock is one of the most common causes of organ dysfunction and death in critically ill patients [2]. Depending on the etiology, the short-term mortality of shock ranges between 30 and 60% [2]. Traditionally, shock has been defined as the presence of arterial hypotension (systolic arterial blood pressure < 90 mmHg or mean arterial blood pressure < 65 mmHg) with or without clinical or laboratory signs of tissue hypoperfusion [2,3,4]. However, these shock definitions have been criticized because they do not include compensated or occult shock states, which are characterized by the presence of tissue hypoperfusion despite normal or even elevated arterial blood pressure values [5,6,7]. Since circulatory dysfunction in this patient group is often masked by preserved macrocirculatory hemodynamic values, the initiation of shock therapy may be delayed, with potentially adverse effects on organ recovery and survival.

In 2014, a task force of the European Society of Intensive Care Medicine (ESICM) recommended that arterial hypotension, although commonly present in patients with shock, should not be required to define shock [1]. Instead, the panel suggested frequent clinical assessments of skin perfusion, urine output, and mental state to identify patients in shock [1].

In recent years, there has been growing interest in using simple bedside clinical signs as indicators and prognostic parameters in both adult and pediatric critical illness [8,9,10,11,12]. However, their predictive value remains debated. Capillary refill time (CRT), for instance, has been shown to correlate well with microvascular blood flow in ICU patients [13] and has been proposed as a valuable triage tool for identifying critically ill patients, especially septic shock patients [14,15]. In contrast, a meta-analysis including 60,656 patients across 23 studies demonstrated only a poor diagnostic ability of CRT to predict death and adverse events in patients at risk or already established acute circulatory failure [16].

A recently published systematic review found that, with an overall moderate risk of bias, reduced peripheral perfusion/temperature, prolonged capillary refill time, skin mottling, and a shock index ≥ 0.7–0.8 were valid clinical indicators of shock [17]. However, scientific evidence to support the use of single clinical signs or a combination thereof to diagnose shock is limited [17,18].

This study aimed to determine the predictive value of eight clinical signs (tachycardia, prolonged capillary refill time, skin mottling, a weak radial pulse, inadequate peripheral perfusion, a shock index > 0.8, altered mental state, and diaphoresis) to identify shock in 317 patients during the early phases of their critical illness.

## 2. Materials and Methods

### 2.1. Study Design and Ethical Approval

This study was designed as a retrospective cohort study and analysis of a systematically collected clinical database. From 1 October 2022, until 30 September 2023, it was conducted in the Critical Care Resuscitation Unit (CCRU) of the Department of Anesthesiology and Critical Care Medicine at the Kepler University Hospital, an 1830-bed tertiary teaching hospital in Linz, Austria. The study protocol was evaluated and approved by the Institutional Ethics Committee (Ethics Committee of the Medical Faculty of Johannes Kepler University Linz, Krankenhausstraße 5, 4020 Linz, Austria; Chairperson: Univ. Prof. Dr. Johannes Fischer) on 27 November 2023 (Reference Number 1298/2023). In view of the retrospective and non-interventional design of the study, the requirement for written informed consent was waived.

### 2.2. Study Setting and Database

The CCRU at the Kepler University Hospital is a spacious room located in close vicinity to the emergency department, radiology department, operating theatre, and a mixed surgical-medical intensive care unit (ICU). It includes two fully equipped ICU patient spaces and is staffed by two critical care nurses and one ICU consultant. The CCRU receives critically ill adult emergency patients who are admitted either by emergency medical services (~15%), the emergency department (~70%), or hospital wards (~15%). The entire spectrum of critical care interventions, ranging from basic respiratory support to extracorporeal cardiopulmonary resuscitation, can be provided in the CCRU. Patients with major trauma are not admitted to the CCRU but to the adjacent trauma bay.

For each patient, a predefined patient data form, which is part of the electronic hospital database, was mandatorily completed for clinical documentation by the ICU consultant responsible for the CCRU. This patient data form systematically collects information on the chief complaint, patient history, premorbid conditions, chronic drug therapies, known allergies, clinical status of the patient at CCRU admission, diagnostic work-up, working diagnosis, critical care interventions provided, patient course, and disposition. For clinical evaluation of the cardiovascular system, the following variables were evaluated and documented in every CCRU patient: heart rhythm, highest heart rate and lowest arterial blood pressure, quality of the radial pulse (strong or weak/faint/non-palpable), peripheral perfusion as assessed by the temperature of the hands (warm to touch/adequate or cold to touch/inadequate), capillary refill time (normal or prolonged; prolonged defined as a time span ≥ 4 s for return of normal skin colour after application of firm pressure to the ventral surface of the distal phalanx of the fingers for 10 s) [19], Mottling Score [20], and presence or absence of diaphoresis during the first hour following CCRU admission.

### 2.3. Patient Population

All patients admitted to the CCRU during the observation period were eligible for enrolment in the study. Patients younger than 18 years were excluded.

### 2.4. Study Variables and Data Extraction

The following variables were extracted from the electronic hospital database in all study patients: age, sex, body mass index (calculated as the body weight in kilograms divided by the body height in metres squared), number and type of comorbidities, chief complaint at CCRU admission, presence and type of shock, the highest heart rate, lowest arterial blood pressure, lowest plethysmographic oxygen saturation, and the most aberrant body temperature measured during the first hour following CCRU admission, presence of tachycardia (highest heart rate > 100 bpm), a pathologic radial pulse (defined as weak, faint or non-palpable), inadequate peripheral perfusion (defined as peripheral temperature of the hands cold to touch), prolonged capillary refill time (defined as ≥4 s), skin mottling (Mottling score ≥ 1), diaphoresis, presence of an altered mental state (defined as the presence of either agitation, somnolence, stupor or coma) during the first hour following CCRU admission, selected critical care interventions delivered during the CCRU stay (continuous vasopressor infusion, blood transfusion, non-invasive or invasive mechanical ventilation), disposition at CCRU discharge, length of ICU and/or hospital stay, and 28-day mortality. In addition, the pH value, lactate level, and base deficit of the first (arterial or venous) blood gas analysis sampled after CCRU admission, as well as the platelet count and serum levels of creatinine and bilirubin determined immediately before (within <1 h) or during the CCRU stay were collected. Using the most aberrant clinical and laboratory values during the CCRU stay, the Simplified Acute Physiology Score II (SAPS II) [21] and Sequential Organ Failure Assessment (SOFA) score [22] were calculated. The shock index [23] was calculated as the quotient between the heart rate and systolic arterial blood pressure, using the most extreme values measured at the same time point during the first hour after CCRU admission.

### 2.5. Definitions

Using data collected during the first hour after CCRU admission, shock was defined as blood lactate levels > 2 mmol/L, which were considered to result from circulatory causes and/or the need for a continuous vasopressor infusion to reverse arterial hypotension (defined as a systolic arterial blood pressure < 90 mmHg or a mean arterial blood pressure < 65 mmHg). The pathophysiological type of shock (hypovolemic, cardiogenic, obstructive, distributive/vasodilatory) was determined based on the clinical diagnosis by the CCRU consultant in charge or the clinical context (e.g., critical analysis and chart review of both the documented course of the patient and discharge diagnosis by the study team). Hypovolemic, cardiogenic, and obstructive shock states were grouped into shock states with low cardiac output.

### 2.6. Study Goals

The primary goal of this study was to determine the predictive value of eight clinical signs (tachycardia, prolonged capillary refill time, skin mottling, a weak radial pulse, inadequate peripheral perfusion, a shock index > 0.8, altered mental state, and diaphoresis), alone or in combination, to identify patients with shock. The secondary study goal was to determine the predictive value of these clinical signs, alone or in combination, to identify patients with shock states with low cardiac output and patients with distributive/vasodilatory shock.

### 2.7. Statistical Analysis

Following data extraction, plausibility checks were performed. These checks included confirmation or elimination of out-of-range values (e.g., extreme outliers). Wherever possible, erroneous variables were re-extracted from the original records and re-entered into an electronic spread sheet. All categorical variables were one-hot encoded. No data imputation methods were applied in case of missing values.

Normal distribution of continuous variables was tested using the Shapiro–Wilk test. Demographic, clinical, laboratory, and outcome data were presented as median values with interquartile ranges or absolute numbers and frequencies. Continuous variables were compared between the patients with and without shock using the Wilcoxon rank-sum test. Categorical variables were compared for group differences using the chi-squared or Fisher’s exact test, as appropriate. Modified bar diagrams were plotted to evaluate the frequency of the eight clinical signs evaluated, alone and in combination, in patients with and without shock. Two-by-two contingency tables were used to calculate the sensitivity, specificity, positive and negative likelihood ratios, as well as the accuracy and odds ratio with 95% confidence intervals to determine the value of the eight clinical signs, alone and in combination, to predict the presence of shock. Additional multivariable logistic regression models were constructed, adjusting for age, sex, and body mass index. Spearman’s rank correlation coefficients (ρ) were applied to test for correlations between clinical signs and SOFA Score as well as lactate levels. Alpha-adjusting to account for multiple testing was performed using the Benjamini–Hochberg false discovery rate method. All statistical analyses were performed using R^®^ statistical software version 4.2.0 (R^®^ Core Development Team, Vienna, Austria). An alpha level of 0.05 was used for all analyses, and all reported hypothesis tests were two-sided. Owing to the exploratory nature of this study, no sample size calculation was performed.

## 3. Results

During the study period, 317 patients were admitted to CCRU. All patients were aged ≥18 years and hence were included in the statistical analysis. The percentage of missing clinical sign data was 4% (Supplementary Material, Figure S1). Table 1 presents the characteristics of all study patients, as well as those with and without shock. Patients with and without shock differed in age, frequency of chronic respiratory disease, chief complaint, selected critical care interventions, initial SOFA score during treatment at the CCRU, need for emergency surgery, disposition at CCRU discharge, and 28-day mortality rate.

Arterial hypotension, gastrointestinal bleeding, sepsis or suspected infection, cardiac arrhythmias, and acute abdomen were significantly more frequently documented as chief complaints in patients with shock. In contrast, respiratory failure, intoxication, and presentations grouped as “other chief complaints” were more commonly observed in patients without shock. The most frequent diagnoses within the “other” category were hyperkalemia (*n* = 6, 1.9%), hyponatremia (*n* = 6, 1.9%), metabolic acidosis (*n* = 6, 1.9%), renal failure (*n* = 6, 1.9%), post-cardiac arrest (*n* = 5, 1.6%), dehydration (*n* = 4, 1.3%), syncope (*n* = 4, 1.3%), and liver failure (*n* = 3, 0.9%).

**Figure 1:**
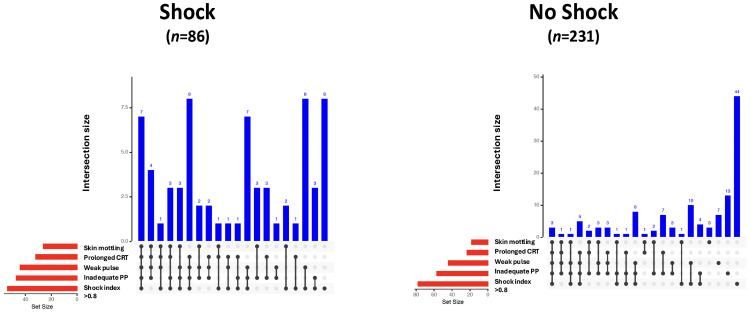
UpSet plots illustrating the frequency and co-occurrence of clinical signs as indicators of shock in patients with (*n* = 86) and without shock (*n* = 231). Vertical blue bars represent the number of patients exhibiting a specific combination of clinical signs (intersection size). The presence of signs within each combination is indicated by black dots and connecting lines. Horizontal red bars show the total number of patients exhibiting each individual clinical sign (set size). CRT, capillary refill time; PP, peripheral perfusion.

Vital parameters, laboratory values, and clinical signs of all study patients, as well as those with and without shock, are given in Table 2. Skin mottling, a weak radial pulse, prolonged capillary refill time, inadequate peripheral perfusion, and a shock index > 0.8 were more frequently observed in patients with shock. The modified bar diagrams of the frequency and combination of clinical signs in patients with and without shock are shown in Figure 1.

### 3.1. Primary Study Goal

**Figure 2:**
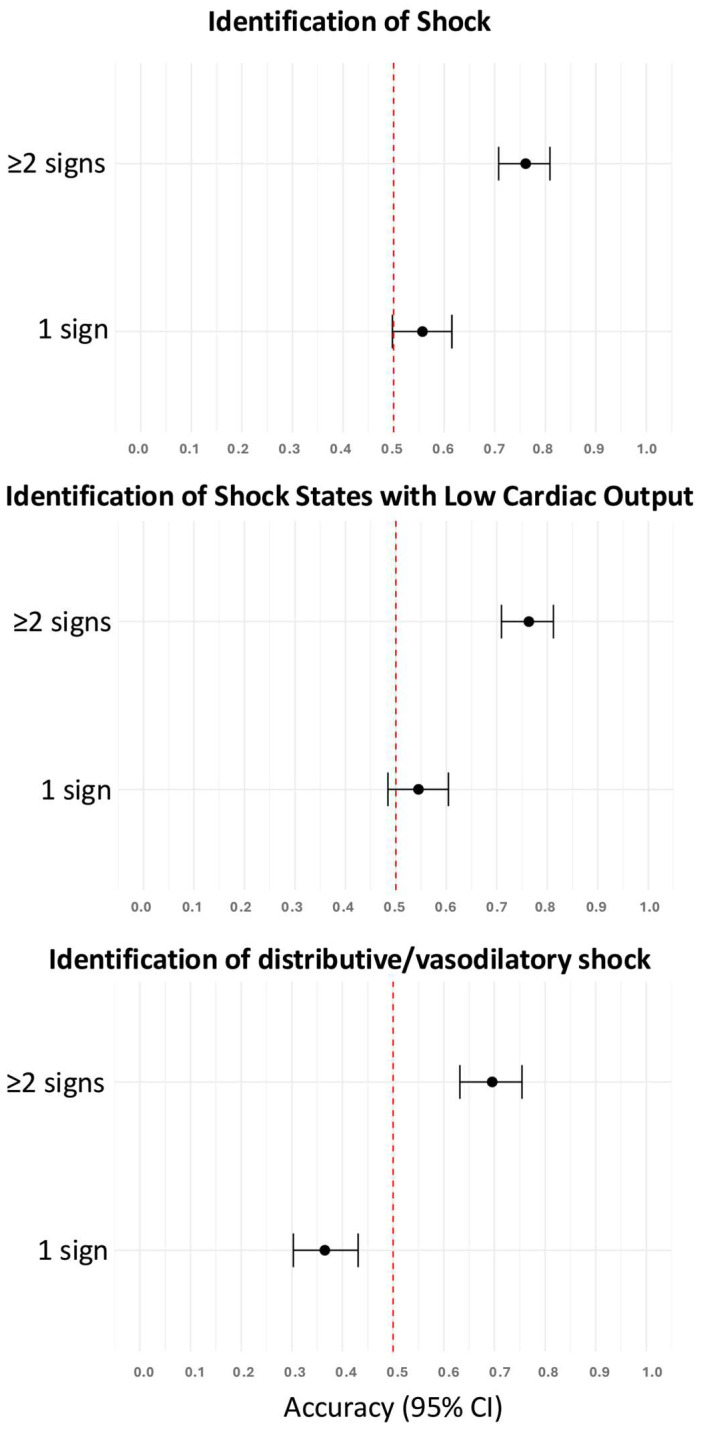
Accuracies of one and two or more clinical signs to identify patients with shock, shock states with low cardiac output, and distributive/vasodilatory shock. The red dotted vertical line indicates the reference accuracy of 0.5 (chance level). CI, confidence interval.

As a single clinical sign, skin mottling, prolonged capillary refill time, shock index > 0.8, weak radial pulse, and inadequate peripheral perfusion were predictive of the presence of shock (Table 3). The accuracies of these clinical signs to identify patients with shock were higher if two or more clinical signs were present compared to only one clinical sign (Figure 2). In a follow-up analysis limited to these five predictive signs, diagnostic accuracy increased progressively with the number of signs observed, plateauing beyond ≥4 signs, thus supporting the additive value of combined clinical assessment (Supplementary Material, Figure S2).

### 3.2. Secondary Study Goals

As a single clinical sign, prolonged capillary refill time, skin mottling, inadequate peripheral perfusion, a weak radial pulse, and a shock index > 0.8 could identify patients with shock states with low cardiac output (Table 3). A shock index > 0.8, tachycardia, and weak radial pulse were predictive of distributive/vasodilatory shock (Table 3). The accuracies of these clinical signs to identify patients with either shock states with low cardiac output or distributive/vasodilatory shock were higher if two or more clinical signs were present compared to only one (Figure 2).

Logistic regression analyses adjusted for age, sex, and body mass index confirmed the overall predictive pattern observed in the univariable analyses. In shock states with low cardiac output, skin mottling, prolonged capillary refill time, weak radial pulse, inadequate peripheral perfusion, tachycardia, and a shock index > 0.8 remained significantly associated with shock occurrence. In contrast, in distributive/vasodilatory shock, associations were weaker and only tachycardia, weak radial pulse, and a shock index > 0.8 reached statistical significance. A detailed summary of adjusted odds ratios, 95% confidence intervals, and *p*-values for all clinical signs (overall shock, low cardiac output shock, and distributive/vasodilatory shock) is provided in Supplementary Table S1.

We further calculated Spearman’s rank correlation coefficients (ρ) to test associations between clinical signs of shock and markers of organ dysfunction and hypoperfusion. Skin mottling (ρ = 0.173, *p* = 0.003), weak radial pulse (ρ = 0.208, *p* < 0.001), inadequate peripheral perfusion (ρ = 0.183, *p* = 0.002), and shock index >0.8 (ρ = 0.122, *p* = 0.033) were significantly correlated with SOFA score, whereas prolonged capillary refill time (ρ = 0.077, *p* = 0.19) was not. All of these clinical signs correlated significantly with plasma lactate levels (skin mottling ρ = 0.27, prolonged capillary refill time ρ = 0.32, weak radial pulse ρ = 0.37, inadequate peripheral perfusion ρ = 0.40, shock index >0.8 ρ = 0.41; all *p* < 0.001).

## 4. Discussion

### 4.1. Summary of Key Findings

In this retrospective study, we aimed to isolate and determine the predictive value of eight predefined clinical signs to identify shock in critically ill patients. Clinical signs are often the first information available during early patient assessment and form the foundation of bedside evaluation. To avoid circular reasoning, we defined shock using objective criteria and explicitly excluded clinical signs from the definition of shock. For this analysis, we therefore defined shock as elevated lactate levels that were considered to result from circulatory causes and/or the need for continuous vasopressor infusion to reverse arterial hypotension. As the presence of shock was the dependent variable in our analysis, its definition was crucial to the results of this study. By combining elevated lactate levels and/or vasopressor dependency, the definition of shock used in this study allowed us to include patients with both compensated/occult and decompensated/overt shock without incorporating clinical signs (the independent variable in this analysis) into the shock definition.

Our study enrolled a general population of critically ill adult patients with moderate to high disease severity (median SAPS II score, 35 points; median SOFA score, 4 points). Except for major trauma, the study patients suffered from a wide range of medical, neurological, and surgical conditions. Accordingly, all four pathophysiological shock types were encountered. As the study variables were collected during the early phases of their critical illness, most patients had not received any or only a few hemodynamic interventions. This may explain why the frequency of distributive/vasodilatory shock in our study was rather low compared with studies that recruited patients only at later stages of their critical illness (e.g., after ICU admission) [24].

Skin mottling, prolonged capillary refill time, a shock index > 0.8, weak radial pulse, and inadequate peripheral perfusion were predictive of shock in this population. The presence of two or more clinical signs had a higher accuracy in identifying patients with shock than the presence of one clinical sign alone. All clinical signs had a high specificity and positive likelihood ratio for predicting shock, while both the sensitivity and negative likelihood ratio were comparatively low. At the bedside, this means that the presence of one and particularly two or more of these clinical signs in a critically ill patient should alert the physician that the patient is at high risk of being in shock, whereas the absence of skin mottling, prolonged capillary refill time, shock index > 0.8, weak radial pulse, or inadequate peripheral perfusion cannot exclude the presence of shock. Interestingly, neither altered mental state, diaphoresis, nor tachycardia was predictive of shock in this population. From a clinical perspective, all the latter signs may be present in patients with shock but are also commonly found in other conditions (e.g., altered mental state in neurological disease or intoxication, diaphoresis in respiratory distress) or general indicators of an increased sympathetic tone (e.g., diaphoresis, tachycardia).

Prolonged capillary refill time, skin mottling, and inadequate peripheral perfusion were predictive of shock states with low cardiac output but not distributive/vasodilatory shock. As all these signs are clinical indicators of reduced skin perfusion or centralization [25], they are physical hallmarks of shock states with low cardiac output. Since distributive/vasodilatory shock is pathophysiologically characterized by peripheral vasodilatation and maldistribution of a normal or increased cardiac output, it is comprehensible that our analysis found that neither skin mottling, prolonged capillary refill time, nor inadequate peripheral perfusion were predictive of the presence of distributive/vasodilatory shock. Instead, a shock index > 0.8 (indicating compensatory tachycardia and/or arterial hypotension), tachycardia, and a weak radial pulse (indicating arterial hypotension), were identified as predictors of the presence of distributive/vasodilatory shock in this population.

### 4.2. Relationship with Previous Studies

Our results are broadly consistent with previous reports while adding important nuances. Capillary refill time has been correlated with lactate levels, sonographic indicators of visceral organ perfusion, presence of shock, and SOFA score count in several studies [14,26,27,28,29]. The findings of this study confirm this association and demonstrate in adjusted analyses, that capillary refill time remains independently predictive of shock. Importantly, its prognostic value was limited to patients with low cardiac output shock, as no association was observed in distributive or vasodilatory shock. This aligns with prior evidence from cardiogenic shock, where prolonged capillary refill time predicted 90-day mortality [30], but differs from findings from Vazquez et al. [28], who reported high sensitivities and specificities for septic/high-output shock. Notably, overall diagnostic accuracies were similar (0.86 vs. 0.79) between studies. Consistent with prior studies [8,14,31], capillary refill time was significantly associated with plasma lactate levels, although, in our study cohort, no correlation with SOFA score was observed.

In our study, skin mottling correlated with both plasma lactate levels and SOFA score. These findings are in line with previously published data, where a strong relationship between skin mottling and lactate levels, urine output, and SOFA score count was established in a landmark study [20] and confirmed by other authors [26,32]. Skin mottling was highly predictive in low cardiac output shock patients, consistent with data from post-cardiac surgery and cardiogenic shock cohorts, where increased mottling scores were highly specific for peripheral hypoperfusion and associated with worse outcome [33,34].

An increased central-to-peripheral temperature gradient or inadequate peripheral perfusion has equally been reported to correlate with lactate levels, the presence of shock, and the SOFA score in critically ill patients [9,10,27,28,29,35]. The findings of our study reinforce these observations by demonstrating an independent association with shock occurrence after adjustment for age, sex and body mass index, as well as significant correlations with both SOFA score and lactate levels.

A large number of studies, primarily conducted in trauma and cardiac patients, reported that a shock index ≥ 0.7–0.8 was predictive of the presence of shock [17,36,37,38]. In our mixed cohort of critically ill adult patients, shock index > 0.8 was strongly predictive for both low cardiac output and distributive/vasodilatory shock states and correlated closely with plasma lactate levels, confirming and extending findings of previous studies [39,40].

To date, only one study has evaluated the association between a nonpalpable radial pulse and mortality in critically ill patients [41]. To the best of our knowledge, no study to date has investigated the relationship between both an altered mental state as well as diaphoresis, and the presence of shock.

### 4.3. Limitations

Important limitations must be considered when interpreting the results of our analysis. First, it was a retrospective study. Although the rate of missing data was very low, and the variables included in the analysis were systematically collected during routine clinical practice, the validity of our results is still inferior to that of a prospective study. Second, this was a single-centre study, which implies that it is unclear whether our findings can be extrapolated to other settings. This is particularly relevant for healthcare settings in which critically ill patients are treated during other or later stages of their illness (e.g., operating theatre or ICU). Finally, the rather wide 95% confidence intervals of our study endpoints indicate that the total number of patients and the number of patients in shock were comparatively low. A larger study population would likely have resulted in more accurate estimates.

Furthermore, data on the presence, extent, and anatomical distribution of peripheral arterial occlusive disease, which may have influenced the interpretation of certain clinical signs such as peripheral pulse quality, CRT or skin temperature, have not been systematically collected and therefore represent a limitation of this study. Since peripheral arterial occlusive disease predominantly affects the lower extremities, we attempted to minimize this potential bias by clinically assessing peripheral perfusion, capillary refill time, and skin temperature at the upper extremities. Nevertheless, we cannot entirely rule out a potential influence of undiagnosed or undocumented arterial disease on our clinical findings.

Another limitation is the absence of reliable data on respiratory rate. Despite being an easily obtainable and clinically important variable, it could not be assessed in our retrospective dataset due to missing documentation. Future studies should incorporate it systematically.

## 5. Conclusions

Skin mottling, prolonged capillary refill time, shock index > 0.8, weak radial pulse, and inadequate peripheral perfusion could identify critically ill patients with shock, particularly shock states with low cardiac output, with a high specificity and positive likelihood ratio. The accuracies of these clinical signs to predict shock were higher if two or more clinical signs were present compared to only one. Larger prospective studies are needed to confirm and extend our findings.

## Figures and Tables

**Table 1:** Characteristics of all study patients as well as study patients categorized by the presence of shock.

		All	Shock	No Shock	*p*-Value
*N*		317	86	231	
**Age**	years	68 (55–78)	69 (63–81)	68 (53–77)	0.03 *
**Male sex**	*n* (%)	193 (60.9%)	56 (65.1%)	137 (59.3%)	0.42
**Body mass index**	kg/m^2^	25.9 (22.5–31.2)	25.3 (22.5–29.4)	26.4 (22.7–32.1)	0.23
**Comorbid conditions**					
*arterial hypertension*	*n* (%)	168 (53%)	45 (52.3%)	123 (53.2%)	0.98
*diabetes mellitus*	*n* (%)	102 (32.2%)	29 (33.7%)	73 (31.6%)	0.82
*chronic respiratory disease*	*n* (%)	93 (29.3%)	14 (16.3%)	79 (34.2%)	0.003 *
*coronary artery disease*	*n* (%)	80 (25.2%)	26 (30.2%)	54 (23.4%)	0.27
*hypercholesterinaemia*	*n* (%)	78 (24.6%)	27 (31.4%)	51 (22.1%)	0.12
**Source of admission**					
*emergency department*	*n* (%)	255 (80.4%)	70 (81.4%)	185 (80.1%)	0.92
*hospital ward*	*n* (%)	62 (19.6%)	16 (18.6%)	46 (19.9%)	
**Chief complaint**					
*respiratory failure*	*n* (%)	85 (26.8%)	13 (15.1%)	72 (31.2%)	0.006 *
*arterial hypotension*	*n* (%)	30 (9.5%)	18 (20.9%)	12 (5.2%)	<0.001 *
*gastrointestinal bleeding*	*n* (%)	26 (8.2%)	13 (15.1%)	13 (5.6%)	0.01 *
*intoxication*	*n* (%)	24 (7.6%)	0 (0%)	24 (10.4%)	<0.001 *
*sepsis/infection*	*n* (%)	24 (7.6%)	12 (14.0%)	14 (6.1%)	0.04 *
*neurological disorder*	*n* (%)	18 (5.7%)	3 (3.5%)	15 (6.5%)	0.42
*heart failure/cardiogenic pulmonary edema*	*n* (%)	15 (4.7%)	5 (5.8%)	10 (4.3%)	0.56
*arrhythmia*	*n* (%)	12 (3.8%)	7 (8.1%)	5 (2.2%)	0.02 *
*acute abdomen*	*n* (%)	10 (3.2%)	7 (8.1%)	3 (1.3%)	0.005 *
*diabetic ketoacidosis*	*n* (%)	8 (2.5%)	2 (2.3%)	6 (2.6%)	1
*others*	*n* (%)	65 (20.5%)	6 (7.0%)	57 (24.7%)	<0.001 *
**Shock criteria**					
*elevated lactate levels due to circulatory causes*	*n* (%)	82 (25.9%)	82 (95.3%)	0 (0%)	
*need for vasopressor infusion*	*n* (%)	49 (15.5%)	49 (57%)	0 (0%)	
**Type of shock**					
*hypovolaemic*	*n* (%)	44 (13.9%)	44 (51.2%)	n.a.	
*cardiogenic*	*n* (%)	26 (8.2%)	26 (30.2%)	n.a.	
*vasodilatory/distributive*	*n* (%)	11 (3.5%)	11 (12.8%)	n.a.	
*obstructive*	*n* (%)	5 (1.6%)	5 (5.8%)	n.a.	
**Critical care interventions**					
*vasopressor infusion*	*n* (%)	49 (15.5%)	49 (57%)	0 (0%)	<0.001 *
*blood transfusion*	*n* (%)	25 (7.9%)	13 (15.1%)	12 (5.2%)	0.007 *
*non-invasive MV*	*n* (%)	90 (28.4%)	16 (18.6%)	74 (32%)	0.03 *
*invasive MV*	*n* (%)	42 (13.2%)	20 (23.3%)	22 (9.5%)	0.003 *
**SAPS II**	points	35 (31–39)	36 (31–42)	34 (30.8–38)	0.26
**SOFA score**	points	4 (2–6)	6 (3.3–9)	3 (2–5)	<0.001 *
**Need for emergency surgery**	*n* (%)	19 (6%)	12 (14%)	7 (3%)	<0.001 *
**Disposition at CCRU discharge**					
*ICU*	*n* (%)	119 (37.5%)	43 (50%)	76 (32.9%)	0.008 *
*hospital ward*	*n* (%)	171 (53.9%)	30 (34.9%)	141 (61%)	<0.001 *
*operating theatre*	*n* (%)	14 (4.4%)	10 (11.6%)	4 (1.7%)	<0.001 *
*discharge*	*n* (%)	10 (3.2%)	0 (0%)	10 (4.3%)	0.07
*died in CCRU*	*n* (%)	3 (0.9%)	3 (3.5%)	0 (0%)	0.02 *
**ICU length of stay** (*n* = 119)	days	5 (3–9)	5 (3–9)	5 (3–9)	0.72
**Hospital length of stay**	days	10 (5–17)	11 (5–18)	9 (5–16)	0.45
**28-day mortality**	*n* (%)	48 (15.1%)	24 (27.9%)	24 (10.4%)	<0.001 *

ICU, intensive care unit; MV, mechanical ventilation; n.a., not applicable; SAPS II, Simplified Acute Physiology Score II; SOFA, Sequential Organ Failure Assessment. *, significant difference between patients with and without shock. Data are given as median values with interquartile range, if not otherwise indicated. Bold indicates main variable categories, italics indicate subcategories.

**Table 2:** Vital parameters, laboratory results, and clinical signs in all study patients as well as study patients categorized by the presence of shock.

		All	Shock	No Shock	*p*-Value
*n*		317	86	231	
**Vital parameters**					
*heart rate*	bpm	102 (84–122)	110 (86–138)	100 (82–120)	0.02 *
*systolic arterial blood pressure*	mmHg	118 (90–150)	90 (76–110)	130 (104–160)	<0.001 *
*mean arterial blood pressure*	mmHg	82 (67–102)	63 (54–78)	90 (73–107)	<0.001 *
*plethysmographic SO_2_*	%	94 (90–96)	95 (91–97)	94 (89–96)	0.24
*body temperature*	°C	36.6 (36–37.2)	36.5 (36–37.2)	36.6 (36–37.2)	0.4
**Laboratory results**					
*pH*		7.33 (7.22–7.4)	7.3 (7.18–7.4)	7.33 (7.25–7.4)	0.26
*base deficit*	mmol/L	−2.4 (−7–0.8)	−6.5 (−11.3–−2.7)	−1.3 (−4.3–1.7)	<0.001 *
*lactate*	mmol/L	2.1 (1.1–4)	4.3 (2.8–6.5)	1.3 (1–2.5)	<0.001 *
*platelets*	G/L	245 (181–313)	252 (179–312)	242 (181–312)	0.96
*creatinine*	mg/dL	1.13 (0.81–1.88)	1.62 (1.07–2.38)	1.04 (0.77–1.48)	<0.001 *
*bilirubin*	mg/dL	0.5 (0.32–0.8)	0.69 (0.42–1.09)	0.43 (0.3–0.73)	<0.001 *
**Clinical signs**					
*tachycardia*	*n* (%)	155 (48.9%)	49 (57%)	106 (45.9%)	0.1
*shock index > 0.8*	*n* (%)	148 (46.7%)	66 (76.7%)	82 (35.5%)	<0.001 *
*altered mental state*	*n* (%)	127 (41.2%)	39 (47%)	88 (39.1%)	0.26
*inadequate peripheral perfusion*	*n* (%)	116 (38.3%)	57 (67.9%)	59 (26.9%)	<0.001 *
*weak/thready radial pulse*	*n* (%)	95 (31.9%)	48 (61.5%)	47 (21.4%)	<0.001 *
*prolonged capillary refill time*	*n* (%)	57 (19.4%)	34 (44.2%)	23 (10.6%)	<0.001 *
*skin mottling*	*n* (%)	49 (16.2%)	31 (37.8%)	18 (8.2%)	<0.001 *
*diaphoresis*	*n* (%)	29 (9.6%)	10 (12.3%)	19 (8.6%)	0.46

SO_2_, oxygen saturation. *, significant difference between patients with and without shock. Data are given as median values with interquartile range, if not otherwise indicated. Bold indicates main variable categories, italics indicate subcategories.

**Table 3:** Predictive value of clinical signs to identify patients with shock, shock states with low cardiac output, and distributive/vasodilatory shock.

**Predictive Value to Identify Shock**							
	**Sensitivity**	**Specificity**	**+LR**	**−LR**	**Accuracy**	**OR (95%CI)**	***p*** **-Value**
Skin mottling	0.38	0.92	4.62	0.68	0.77	6.82 (3.54–13.16)	<0.001 *
Prolonged capillary refill time	0.44	0.89	4.17	0.62	0.78	6.67 (3.57–12.45)	<0.001 *
Shock index > 0.8	0.77	0.64	2.15	0.36	0.68	5.96 (3.37–10.52)	<0.001 *
Weak radial pulse	0.62	0.79	2.88	0.49	0.74	5.89 (3.37–10.30)	<0.001 *
Inadequate peripheral perfusion	0.68	0.73	2.52	0.44	0.72	5.73 (3.31–9.89)	<0.001 *
Tachycardia	0.57	0.54	1.24	0.80	0.55	1.56 (0.95–2.57)	0.10
Diaphoresis	0.12	0.91	1.43	0.96	0.70	1.49 (0.66–3.36)	0.46
Altered mental state	0.47	0.61	1.20	0.87	0.57	1.38 (0.83–2.29)	0.26
**Predictive Value to Identify Shock States with Low Cardiac Output**							
	**Sensitivity**	**Specificity**	**+LR**	**−LR**	**Accuracy**	**OR (95%CI)**	***p*** **-Value**
Prolonged capillary refill time	0.48	0.89	4.50	0.58	0.80	7.71 (4.04–14.71)	<0.001 *
Skin Mottling	0.40	0.92	4.92	0.65	0.79	7.57 (3.86–14.85)	<0.001 *
Inadequate peripheral perfusion	0.71	0.73	2.64	0.39	0.73	6.72 (3.73–12.09)	<0.001 *
Weak radial pulse	0.63	0.79	2.93	0.47	0.75	6.18 (3.43–11.16)	<0.001 *
Shock index >0.8	0.76	0.64	2.13	0.37	0.67	5.72 (3.15–10.36)	<0.001 *
Altered mental state	0.47	0.61	1.19	0.88	0.57	1.36 (0.8–2.31)	0.32
Tachycardia	0.53	0.54	1.16	0.86	0.54	1.35 (0.8–2.27)	0.32
Diaphoresis	0.11	0.91	1.30	0.97	0.72	1.34 (0.56–3.22)	0.67
**Predictive Value to Identify Distributive/Vasodilatory Shock**							
	**Sensitivity**	**Specificity**	**+LR**	**−LR**	**Accuracy**	**OR (95%CI)**	***p*** **-Value**
Shock index > 0.8	0.82	0.64	2.29	0.28	0.65	8.12 (1.71–38.49)	<0.01 *
Tachycardia	0.82	0.54	1.78	0.34	0.55	5.31 (1.12–25.1)	0.04 *
Weak radial pulse	0.55	0.79	2.55	0.58	0.77	4.42 (1.29–15.11)	0.03 *
Skin mottling	0.20	0.92	2.44	0.87	0.89	2.81 (0.55–14.22)	0.47
Diaphoresis	0.20	0.91	2.32	0.88	0.88	2.64 (0.52–13.36)	0.51
Inadequate peripheral perfusion	0.45	0.73	1.69	0.75	0.72	2.26 (0.66–7.68)	0.32
Prolonged capillary refill time	0.20	0.89	1.89	0.89	0.86	2.11 (0.42–10.54)	0.68
Altered mental state	0.50	0.61	1.28	0.82	0.60	1.56 (0.44–5.53)	0.72

CI, confidence interval; +LR, positive likelihood ratio; −LR, negative likelihood ratio; OR, odds ratio; *, statistical significance at *p* < 0.05.

## Data Availability

The data are not publicly available because of GDPR and local regulatory/data privacy provisions but are available from the corresponding author (M.N.) upon reasonable request.

## Supplementary Materials

The following supporting information can be downloaded at: https://www.mdpi.com/article/10.3390/diagnostics15172252/s1, Figure S1. Missingness Map of data regarding clinical signs to detect shock; Figure S2. Diagnostic accuracy for shock identification across increasing numbers of clinical signs. The red dotted vertical line indicates the reference accuracy of 0.5 (chance level); CI: confidence interval; Table S1. Predictive value of clinical signs to identify patients with shock, shock states with low cardiac output, and distributive/vasodilatory shock adjusted for age, sex and body mass index. CI: confidence interval; OR: odds ratio; ^§^: alpha-adjusted *p*-values, *: statistical significance at *p* < 0.05.

## Abbreviations

The following abbreviations are used in this manuscript:

CCRU	critical care resuscitation unit
ICU	intensive care unit
LR+	positive likelihood ratio
LR−	negative likelihood ratio
SAPS II	Simplified Acute Physiology score II^12^
SOFA	Sequential Organ Failure Assessment

## References

[1] Cecconi M., De Backer D., Antonelli M., Beale R., Bakker J., Hofer C., Jaeschke R., Mebazaa A., Pinsky M.R., Teboul J.L. (2014). Consensus on circulatory shock and hemodynamic monitoring. Task force of the European Society of Intensive Care Medicine. Intensive Care Med..

[2] Vincent J.-L., De Backer D. (2013). Circulatory Shock. N. Engl. J. Med..

[3] Singer M., Deutschman C.S., Seymour C.W., Shankar-Hari M., Annane D., Bauer M., Bellomo R., Bernard G.R., Chiche J.-D., Coopersmith C.M. (2016). The Third International Consensus Definitions for Sepsis and Septic Shock (Sepsis-3). JAMA.

[4] Baran D.A., Grines C.L., Bailey S., Burkhoff D., Hall S.A., Henry T.D., Hollenberg S.M., Kapur N.K., O’Neill W., Ornato J.P. (2019). SCAI Clinical Expert Consensus Statement on the Classification of Cardiogenic Shock: This Document Was Endorsed by the American College of Cardiology (ACC), the American Heart Association (AHA), the Society of Critical Care Medicine (SCCM), and the Society of Thoracic Surgeons (STS) in April 2019. Catheter. Cardiovasc. Interv..

[5] Martin J.T., Alkhoury F., O’Connor J.A., Kyriakides T.C., Bonadies J.A. (2010). ‘Normal’ vital signs belie occult hypoperfusion in geriatric trauma. Am. Surg..

[6] Meregalli A., Oliveira R.P., Friedman G. (2004). Occult hypoperfusion is associated with increased mortality in hemodynamically stable, high-risk, surgical patients. Crit. Care.

[7] Howell M.D., Donnino M., Clardy P., Talmor D., Shapiro N.I. (2007). Occult hypoperfusion and mortality in patients with suspected infection. Intensive Care Med..

[8] Lima A., Jansen T.C., van Bommel J., Ince C., Bakker J. (2009). The prognostic value of the subjective assessment of peripheral perfusion in critically ill patients. Crit. Care Med..

[9] Amson H., Vacheron C.-H., Thiolliere F., Piriou V., Magnin M., Allaouchiche B. (2020). Core-to-skin temperature gradient measured by thermography predicts day-8 mortality in septic shock: A prospective observational study. J. Crit. Care.

[10] Mongkolpun W., Orbegozo D., Cordeiro C.P.R., Franco C.J.C.S., Vincent J.-L., Creteur J. (2020). Alterations in Skin Blood Flow at the Fingertip Are Related to Mortality in Patients With Circulatory Shock. Crit. Care Med..

[11] Walker S.B., Conlon T.W., Zhang B., Mensinger J.L., Fitzgerald J.C., Himebauch A.S., Glau C., Nishisaki A., Ranjit S., Nadkarni V. (2020). Clinical Signs to Categorize Shock and Target Vasoactive Medications in Warm Versus Cold Pediatric Septic Shock. Pediatr. Crit. Care Med..

[12] Kattan E., Hernández G. (2021). The role of peripheral perfusion markers and lactate in septic shock resuscitation. J. Intensive Med..

[13] Morin A., Missri L., Urbina T., Bonny V., Gasperment M., Bernier J., Baudel J.L., Kattan E., Maury E., Joffre J. (2025). Relationship between skin microvascular blood flow and capillary refill time in critically ill patients. Crit. Care.

[14] Ait-Oufella H., Bige N., Boelle P.Y., Pichereau C., Alves M., Bertinchamp R., Baudel J.L., Galbois A., Maury E., Guidet B. (2014). Capillary refill time exploration during septic shock. Intensive Care Med..

[15] Hariri G., Joffre J., Leblanc G., Bonsey M., Lavillegrand J.R., Urbina T., Guidet B., Maury E., Bakker J., Ait-Oufella H. (2019). Narrative review: Clinical assessment of peripheral tissue perfusion in septic shock. Ann. Intensive Care.

[16] Jacquet-Lagrèze M., Pernollet A., Kattan E., Ait-Oufella H., Chesnel D., Ruste M., Schweizer R., Allaouchiche B., Hernandez G., Fellahi J.L. (2023). Prognostic value of capillary refill time in adult patients: A systematic review with meta-analysis. Crit. Care.

[17] Tschoellitsch T., Noitz M., Türk M., Meier J., Dünser M.W. (2023). The value of clinical signs as indicators of shock. Intensive Care Med..

[18] Hiemstra B., Eck R.J., Keus F., van der Horst I.C.C. (2017). Clinical examination for diagnosing circulatory shock. Curr. Opin. Crit. Care.

[19] Hernández G., Ospina-Tascón G.A., Damiani L.P., Estenssoro E., Dubin A., Hurtado J., Friedman G., Castro R., Alegría L., Teboul J.-L. (2019). Effect of a Resuscitation Strategy Targeting Peripheral Perfusion Status vs Serum Lactate Levels on 28-Day Mortality Among Patients With Septic Shock. JAMA.

[20] Ait-Oufella H., Lemoinne S., Boelle P.Y., Galbois A., Baudel J.L., Lemant J., Joffre J., Margetis D., Guidet B., Maury E. (2011). Mottling score predicts survival in septic shock. Intensive Care Med..

[21] Le Gall J.R., Lemeshow S., Saulnier F. (1993). A new Simplified Acute Physiology Score (SAPS II) based on a European/North American multicenter study. JAMA.

[22] Moreno R., Vincent J.-L., Matos R., Mendonça A., Cantraine F., Thijs L., Takala J., Sprung C., Antonelli M., Bruining H. (1999). The use of maximum SOFA score to quantify organ dysfunction/failure in intensive care. Results of a prospective, multicentre study. Intensive Care Med..

[23] Koch E., Lovett S., Nghiem T., Riggs R.A., Rech M.A. (2019). Shock index in the emergency department: Utility and limitations. Open Access Emerg. Med..

[24] De Backer D., Biston P., Devriendt J., Madl C., Chochrad D., Aldecoa C., Brasseur A., Defrance P., Gottignies P., Vincent J.-L. (2010). Comparison of dopamine and norepinephrine in the treatment of shock. N. Engl. J. Med..

[25] Ait-Oufella H., Bourcier S., Alves M., Galbois A., Baudel J.-L., Margetis D., Bige N., Offenstadt G., Maury E., Guidet B. (2013). Alteration of skin perfusion in mottling area during septic shock. Ann. Intensive Care.

[26] Brunauer A., Koköfer A., Bataar O., Gradwohl-Mathis I., Dankl D., Bakker J., Dünser M.W. (2016). Changes in peripheral perfusion relate to visceral organ perfusion in early septic shock: A pilot study. J. Crit. Care.

[27] Filho R.R., de Freitas Chaves R.C., Assunção M.S.C., Neto A.S., De Freitas F.M., Romagnoli M.L., Silva E., Lattanzio B., Dubin A., Corrêa T.D. (2020). Assessment of the peripheral microcirculation in patients with and without shock: A pilot study on different methods. J. Clin. Monit. Comput..

[28] Vazquez R., Gheorghe C., Kaufman D., Manthous C.A. (2010). Accuracy of bedside physical examination in distinguishing categories of shock: A pilot study. J. Hosp. Med..

[29] Hernandez G., Pedreros C., Veas E., Bruhn A., Romero C., Rovegno M., Neira R., Bravo S., Castro R., Kattan E. (2012). Evolution of peripheral vs metabolic perfusion parameters during septic shock resuscitation. A clinical-physiologic study. J. Crit. Care.

[30] Merdji H., Curtiaud A., Aheto A., Studer A., Harjola V.P., Monnier A., Duarte K., Girerd N., Kibler M., Ait-Oufella H. (2022). Performance of Early Capillary Refill Time Measurement on Outcomes in Cardiogenic Shock: An Observational, Prospective Multicentric Study. Am. J. Respir. Crit. Care Med..

[31] Huang W., Xiang H., Hu C., Wu T., Zhang D., Ma S., Hu B., Li J. (2023). Association of Sublingual Microcirculation Parameters and Capillary Refill Time in the Early Phase of ICU Admission. Crit. Care Med..

[32] Ferraris A., Bouisse C., Thiollière F., Piriou V., Allaouchiche B. (2020). Mottling Incidence and Mottling Score According to Arterial Lactate Level in Septic Shock Patients. Indian J. Crit. Care Med..

[33] Luo J.C., Luo M.H., Zhang Y.J., Liu W.J., Ma G.G., Hou J.Y., Su Y., Hao G.W., Tu G.W., Luo Z. (2024). Skin mottling score assesses peripheral tissue hypoperfusion in critically ill patients following cardiac surgery. BMC Anesthesiol..

[34] Merdji H., Bataille V., Curtiaud A., Bonello L., Roubille F., Levy B., Lim P., Schneider F., Khachab H., Dib J.C. (2023). Mottling as a prognosis marker in cardiogenic shock. Ann. Intensive Care.

[35] Bourcier S., Pichereau C., Boelle P.-Y., Nemlaghi S., Dubée V., Lejour G., Baudel J.-L., Galbois A., Lavillegrand J.-R., Bigé N. (2016). Toe-to-room temperature gradient correlates with tissue perfusion and predicts outcome in selected critically ill patients with severe infections. Ann. Intensive Care.

[36] Caputo N., Reilly J., Kanter M., West J. (2018). A retrospective analysis of the respiratory adjusted shock index to determine the presence of occult shock in trauma patients. J. Trauma Acute Care Surg..

[37] Jentzer J.C., van Diepen S., Barsness G.W., Henry T.D., Menon V., Rihal C.S., Naidu S.S., Baran D.A. (2019). Cardiogenic Shock Classification to Predict Mortality in the Cardiac Intensive Care Unit. J. Am. Coll. Cardiol..

[38] El-Menyar A., Al Habib K.F., Zubaid M., Alsheikh-Ali A.A., Sulaiman K., Almahmeed W., Amin H., AlMotarreb A., Ullah A., Suwaidi J.A. (2020). Utility of shock index in 24,636 patients presenting with acute coronary syndrome. Eur. Heart J. Acute Cardiovasc. Care.

[39] Berger T., Green J., Horeczko T., Hagar Y., Garg N., Suarez A., Panacek E., Shapiro N. (2013). Shock index and early recognition of sepsis in the emergency department: Pilot study. West J. Emerg. Med..

[40] Baser M.R., Ganta R., Neeradi C., Varatharajan S. (2025). Serum Lactate Levels and Their Correlation With Hospital Outcomes in ICU Patients With Shock: A Cross-Sectional Study at a Tertiary Care Center. Cureus.

[41] Poloujadoff M.-P., Lapostolle F., Lockey D., Amathieu R., Merouani M., Galinski M., Adnet F. (2006). Survival of severely shocked patients who present with absent radial pulse and unrecordable blood pressure in the pre-hospital phase. Resuscitation.

