# Supplementary material for: The Predictive Value of Clinical Signs to Identify Shock in Critically Ill Patients
Source: Diagnostics (Basel). 2025 Sep 5;15(17):2252. doi: 10.3390/diagnostics15172252 (PMC12428204; doi:10.3390/diagnostics15172252)
Supplement: Supplementary file 1 [file diagnostics-15-02252-s001.zip › Supplementary_Files/Supplementary_Table_S1.docx]

**Supplementary Table S1.** Predictive value of clinical signs to identify patients with shock, shock states with low cardiac output, and distributive/vasodilatory shock adjusted for age, sex and body mass index.

| **Predictive Value to Identify Shock:** |  |  |
| --- | --- | --- |
|  | **Adjusted OR (95%CI)** | ***p*-value^§^** |
| **Skin mottling** | 5.82 (2.85–12.21) | <0.001* |
| **Prolonged capillary refill time** | 5.11 (2.59–10.27) | <0.001* |
| **Shock index >0.8** | 7.46 (3.96–14.74) | <0.001* |
| **Weak radial pulse** | 6.63 (3.52–12.87) | <0.001* |
| **Inadequate peripheral perfusion** | 4.59 (2.55–8.43) | <0.001* |
| **Tachycardia** | 1.62 (0.93–2.86) | 0.09 |
| **Diaphoresis** | 1.50 (0.59–3.58) | 0.37 |
| **Altered mental state** | 1.44 (0.81–2.56) | 0.21 |
|  |  |  |
| **Predictive Value to Identify Shock States with Low Cardiac Output:** |  |  |
|  | **Adjusted OR (95%CI)** | ***p*-value^§^** |
| **Prolonged capillary refill time** | 3.88 (1.35–10.85) | 0.01* |
| **Skin Mottling** | 5.88 (2.02–16.81) | <0.001* |
| **Inadequate peripheral perfusion** | 3.92 (1.55–10.28) | 0.004* |
| **Weak radial pulse** | 3.30 (1.20–8.93) | 0.02* |
| **Shock index >0.8** | 6.69 (2.56–19.95) | <0.001* |
| **Altered mental state** | 1.35 (0.52–3.41) | 0.524 |
| **Tachycardia** | 2.88 (1.16–7.68) | 0.03* |
| **Diaphoresis** | 1.46 (0.31–5.12) | 0.584 |
|  |  |  |
| **Predictive Value to Identify Distributive/Vasodilatory Shock:** |  |  |
|  | **Adjusted OR (95%CI)** | ***p*-value^§^** |
| **Shock index >0.8** | 15.53 (2.71–293.94) | 0.01* |
| **Tachycardia** | 8.91 (1.58–167.47) | 0.04* |
| **Weak radial pulse** | 5.23 (1.34–22.52) | 0.02* |
| **Skin mottling** | 3.54 (0.47–18.44) | 0.16 |
| **Diaphoresis** | 3.25 (0.41–17.79) | 0.20 |
| **Inadequate peripheral perfusion** | 2.62 (0.69–9.99) | 0.15 |
| **Prolonged capillary refill time** | 2.22 (0.31–10.55) | 0.35 |
| **Altered mental state** | 2.04 (0.51–8.66) | 0.31 |

CI, confidence interval; OR, odds ratio.

^§^, alpha-adjusted p-values; *, statistical significance at p < 0.05.
