# Supplementary material for: The Predictive Value of Clinical Signs to Identify Shock in Critically Ill Patients
Source: Diagnostics (Basel). 2025 Sep 5;15(17):2252. doi: 10.3390/diagnostics15172252 (PMC12428204; doi:10.3390/diagnostics15172252)

Identification of Shock

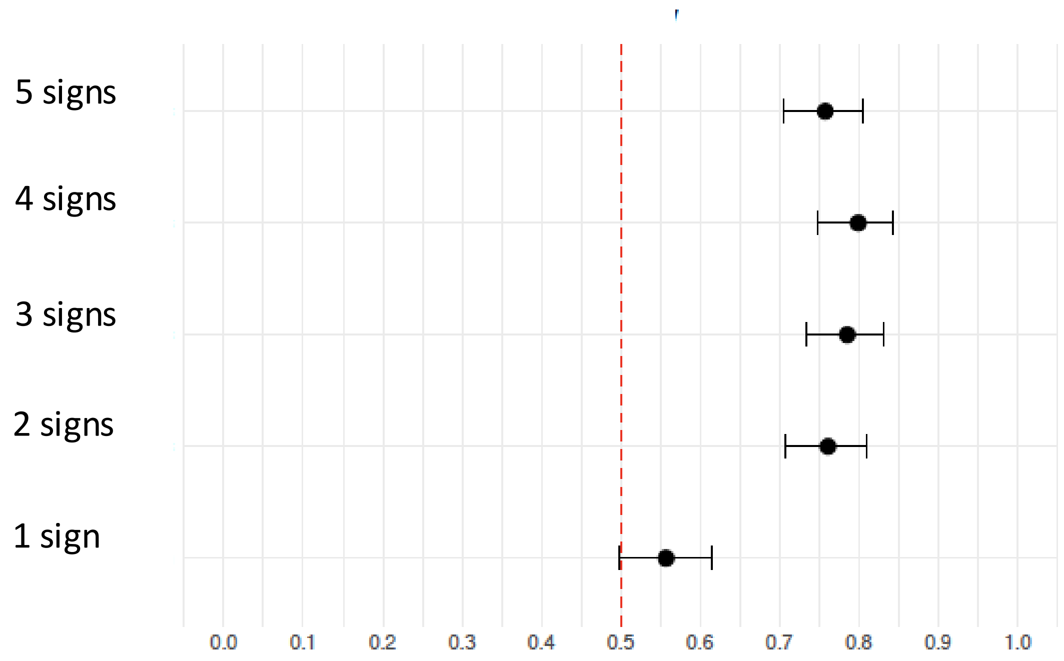

Identification of Shock States with Low Cardiac Output

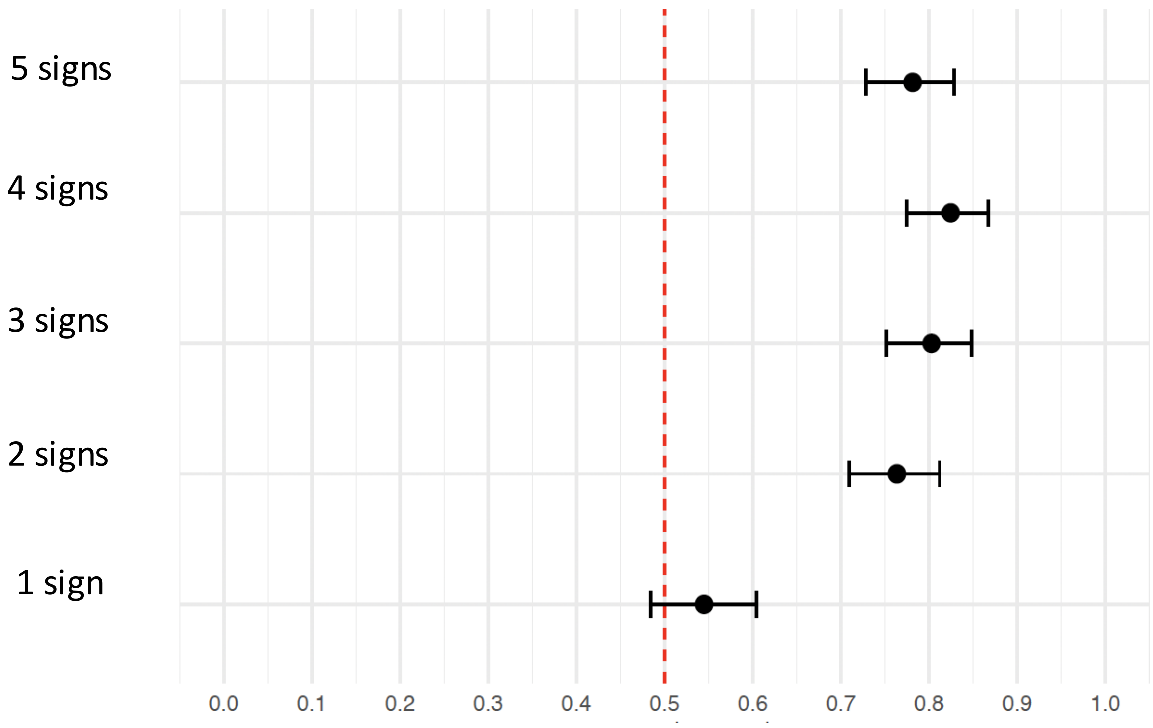

Identification of distributive/vasodilatory shock

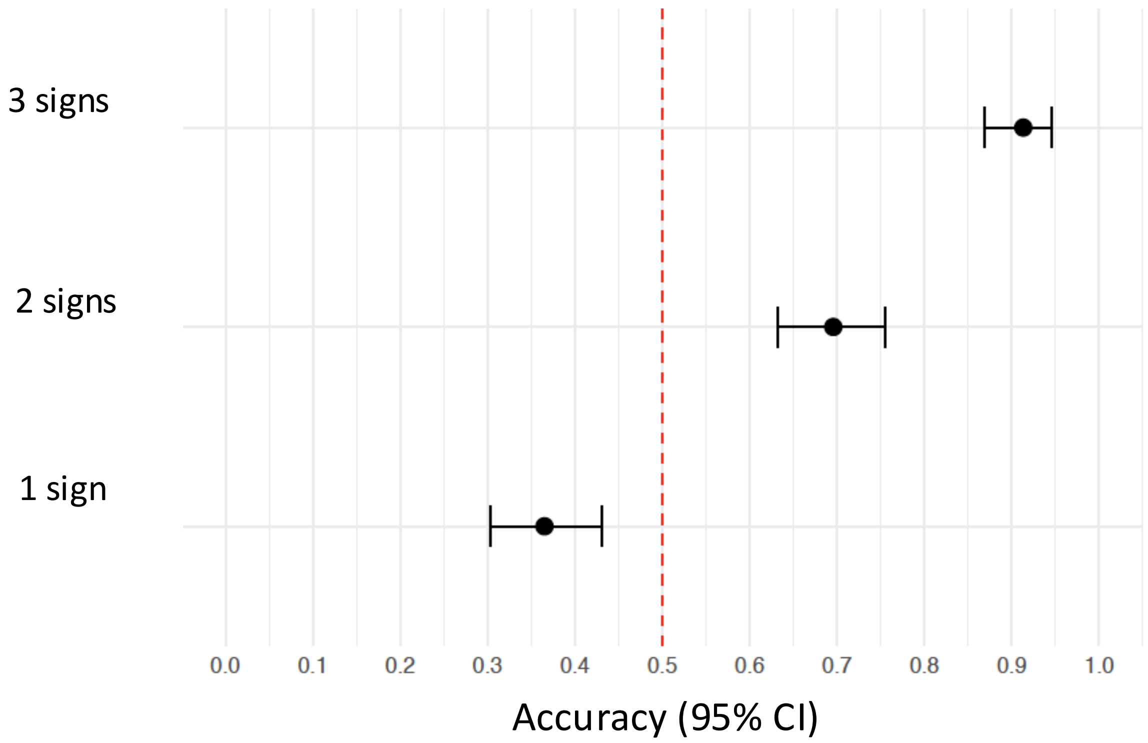

Supplement: Supplementary file 1 [file diagnostics-15-02252-s001.zip › Supplementary_Files/Supplementary_Figure_S2.pdf]
